# Shift work is associated with an increased risk of type 2 diabetes and elevated RBP4 level: cross sectional analysis from the OHSPIW cohort study

**DOI:** 10.1186/s12889-023-16091-y

**Published:** 2023-06-14

**Authors:** Li Wang, Qi Ma, BinBin Fang, YinXia Su, Wanxian Lu, Mengdi Liu, Xue Li, Jiwen Liu, LiJuan He

**Affiliations:** 1 Departments of Public Health, Xinjiang Medical University, Urumqi, 830011 China; 2 State Key Laboratory of Pathogenesis, Prevention and Treatment of High Incidence Diseases in Central Asia, Clinical Medical Research Institute, The First Affiliated Hospital of Xinjiang Medical University, Urumqi, 830011 China; 3 College of Medical Engineering and Technology, Xinjiang Medical University, Urumqi, 830011 China

**Keywords:** Shift work, Type 2 diabetes, Retinol binding protein 4, Sleep quality, Oil workers

## Abstract

**Background:**

Shift work, with its growing prevalence globally, disrupts the body's inherent circadian rhythm. This disruption may escalate the risk of chronic diseasesxacerbate chronic disease risk by dysregulating physiological, behavioral, and psychosocial pathways. This study aimed to evaluate the effect of shift work on type 2 diabetes (T2DM) and Retinol binding protein 4 (RBP4) level.

**Methods:**

The current study employed a multi-stage stratified cluster sampling technique, examining 1499 oilfield workers from the OHSPIW cohort who participated in occupational health assessments between March 2017 and June 2018.The evaluation involved shift work, sleep quality, T2DM status with questionnaires and plasma RBP4 levels in blood samples. Statistical analysis includes, Chi-square tests, t-tests, multivariate logistic regression analyses, and multivariate linear mixed models.

**Results:**

The prevalence rate of T2DM in shift workers (6.56%) was significantly higher than in day workers (4.21%) (OR = 1.60, 95% CI: 1.01–2.53), with no significant difference found in the family history of diabetes, hypertension, or other chronic heart diseases (*P* = 0.378). The shift worker (6.89 ± 3.35) also exhibited distinctly higher PSQI scores than day workers (5.99 ± 2.87) (*P* < 0.001). Adjusting the age, gender, BMI, family income, tobacco smoking, alcohol drinking and PSQI, hailed shift work as a risk factor for T2DM (OR = 1.91, 95% CI: 1.17–3.14). The pairwise comparison revealed significant differences in RBP4 levels across different groups: shift and non-shift workers both with and without T2DM (*P* < 0.001). The RBP4 level of the shift group without T2DM was higher than the non-shift group without T2DM (*P* < 0.05). The levels of RBP4 level in shift and non-shift groups with T2DM was higher than those without T2DM (*P* < 0.05). The multivariate linear mixed model showed that when age, gender, BMI, diabetes, PSQI, family income, smoking and drinking remained unchanged, the RBP4 level of the shift workers increased by an average of 9.51 μg/mL compared with the day workers.

**Conclusions:**

Shift work is associated with an increased risk of T2DM and high levels of RBP4. Follow-up of RBP4 could facilitateearly detection of T2DM among shift workers.

**Supplementary Information:**

The online version contains supplementary material available at 10.1186/s12889-023-16091-y.

## Background

Shift work generally means an organization of working time which is any arrangement of daily working hours other than the standard daylight hours (7–8 am to 5–6 pm). The most common shift schedules include two or three, irregular, or continuous night shifts. Shift work is becoming more common worldwide with the start of modernization and the growing demand for round the clock health, food services, and transportation. It is estimated that 20–30% of full-time workers in Europe, the United States [1, 2], and Japan [3] adhere to alternate shifts, and these numbers continue to rise. Shift work disrupts body's normal circadian rhythm. The International Agency for Research on Cancer (IARC) classified shift work as a group 2A carcinogen in 2007 [4]. The disruption of circadian rhythms can increase the risk of many chronic diseases, such as cancer [4], metabolic disorders [5], cardiovascular diseases [6] and mental health disorders [7] through the dysregulation of normal physiological, behavioral, and psychosocial pathways.

The increasing prevalence of T2DM presents a major global public health challenge, especially in China [8]. Active lifestyle interventions play an important role in the prevention and control of diabetes. However, research exploring shift work's impact on diabetes remains scarce. There were inconsistent research conclusions about whether shift work increased the risk of T2DM. While one cohort study involving 19,837 female nurses reported that night shifts increased the risk of T2DM [9], another study found no statistical correlation between shift work and diabetes [10]. Supplementing evidence of the association between the work environment and diabetes is essential for designing targeted primary and secondary prevention strategies.

Retinol binding protein 4 (RBP4) is a carrier for retinol from the liver to the target organ produced and secreted primarily from the liver [11]. Elevated serum RBP4, an adipokine associated with insulin resistance(IR), stimulate an inflammatory response, impairing insulin sensitivity and involvement in IR. Notably, RBP4 single-nucleotide polymorphism (IRS) has been identified as a risk factor for T2DM [11], making it a promising drug target for T2DM. It has been reported that elevation of RBP4 induced IR in mice [12]. Serum RBP4 levels had a positive relationship with the degree of IR in humans, and sports training reduced RBP4 levels and enhanced insulin sensitivity [13]. Clinical studies have shown that individuals with impaired glucose tolerance and T2DM have elevated circulating RBP4 levels, which was inversely associated with insulin sensitivity [12–15]. However, this conclusion is still controversial, as several studies found no associations between serum RBP4 levels and impaired glucose tolerance, IR, or T2DM [13, 14]. In addition, it was reported [16] that the expression of the RBP4 gene in subcutaneous adipocytes was lower in obese individuals than in their lean counterparts.

RBP4 has the capacity to interact with all links in the insulin receptor/insulin receptor substrate (IRS) / P13K / Ras / mitogen-activated protein kinase (MAPK) signaling pathways. This interaction is accomplished through the activation of c-Jun N-terminal kinase(JNK), NF- κ B inhibits IKB kinase, extracellular signal-regulated kinase, and cytokine signal transduction inhibitor 3. Such activation and inhibition alter downstream gene expression and signal transduction. These changes may ultimately prompt IR and expedite the onset of T2DM [17–20]. A recent study showed that RBP4 had a circadian rhythm in the liver and plasma. As a liver factor, RBP4 plays a circadian role in regulating glucose metabolism, which is necessary for the normal circadian rhythm of insulin sensitivity [21]. Therefore, these findings suggest a potential role of RBP4 in the pathogenesis of T2D in shift workers. Furthermore, RBP4 may serve as a potential biomarker for the inception of IR. In this study, we evaluated the relationship between shift work and the risk of T2DM and RBP4 levels in oil workers in Xinjiang.

While RBP4 is understood to cause IR and T2DM, little is known regarding the relationships among plasma RBP4 levels, shift work and T2DM. Here, we used data from workers' health examinations conducted by a oil company in Xinjiang, China to investigate the association between shift work, T2DM and RBP4 levels.

## Methods

### Study population

**Fig. 1:**
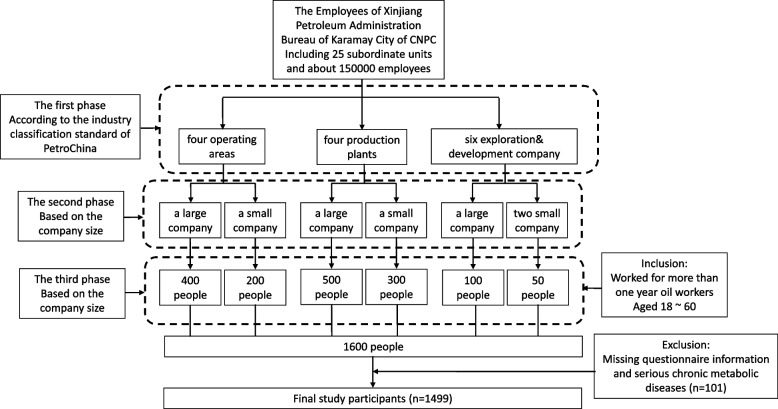
Participant inclusion flowchart

This cross-sectional and descriptive-analytic study was conducted between March 2017 and June 2018. The study focused on the Occupational Health Study of Petroleum Industry Workers(OHSPIW) cohort of petroleum workers who regularly underwent health checkups at the Occupational Health Examination Department of the Central Hospital of Karamay, Xinjiang. The target population encompassed all employees of the Xinjiang Petroleum Administration Bureau of the Karamay City of China National Petroleum Corporation (CNPC). The administration has 25 subordinate units and about 150,000 employees, covering all work associated with the petroleum industry. The three-stage stratified sampling method was adopted, and four operating areas, four production plants and six exploration& development companies were chosen according to the industry classification standard of PetroChina. Based on the company size, a large (> 400 employees) and a small company (< 400 employees) were randomly selected from the operation area. Subsequently, a sample of 400 individuals was drawn from the larger company, while 200 were selected from the smaller one. We executed a comprehensive sampling strategy from both a large (> 1000 workers) and a small manufacturing plant (< 1000 workers), randomly selecting 500 and 300 employees, respectively. One large company (> 200 workers) and two small companies (< 200 workers) were sampled from the exploration& development company, and 100 and 50 people were randomly selected, respectively, then 100 and 50 people were extracted according to the company size. The SPSS software was utilized to randomly sample individuals fulfilling the inclusion and exclusion criteria. The inclusion criteria include oil workers aged 18–60, who have been employed for over a year. Exclusion criteria include workers with missing questionnaire information and serious chronic metabolic diseases such as a history of cancer. The questionnaires were distributed to the respondents as part of the occupational health examination of oil workers, and their blood samples were collected. From a total of 1600 questionnaires distributed, we received 1499 valid responses, with an effective recovery rate of 93.75%. The participant inclusion flowchart is shown in Fig. 1. Subsequently, 157 cases of non-shift and shift workers with and without T2DM were randomly selected for RBP4 expression level test. All procedures were approved by the ethics committee of the First Affiliated Hospital of Xinjiang Medical University and performed in accordance with the Declaration of Helsinki. All participants voluntarily provided written informed consent before the investigation.

### Shift work

The information on shift work was acquired through questionnaire survey. The participants were segregated into two groups: day workers and shift workers. Day workers were those who maintained a work schedule from 09:00 to 20:00, had not been exposed to shift work in the past 3 years, worked in shift work schedules for no more than 6 months in their lifetime, and had no trans menstrual travel (span more than one time zone) in the three months prior to the study. On the other hand, shift workers were those involved in varying work schedules, including two (day/night, 12 h/shift), three (day/evening/night, 8 h/shift), four shifts (divided 24 h a day into four shifts) per day and others. These shift workers were subjected to light-at-night exposure (working a minimum of 6 h between 9 p.m. and 9 a.m.), with at least 3-night shifts per month for a minimum of one year prior to the study.

### Sleep disorders

The Pittsburgh Sleep Quality Index (PSQI) was used to evaluate the subjects' sleep quality over the past month. This tool encompasses seven subscales: subjective sleep quality, sleep latency, sleep duration, habitual sleep efficiency, sleep disturbances, usage of sleep medication, and daytime dysfunction. Each subscale is scored from 0–3, leading to a total score range of 0–21, with higher scores indicating poorer sleep quality. A cumulative score above 7 was defined as indicative of a sleep disorder.

### Determination of T2DM cases

Participants who self-reported T2DM were asked to provide written consent for medical record acquisition. Study researchers blinded to exposure status reviewed medical records to confirm all possible T2DM cases. T2DM cases were defined by the diagnostic criteria for T2DM described by the World Health Organization WHO 1999 (Classic diabetes symptoms) plus any of the following: 1) fasting blood glucose ≥ 7.0 mmol/L; 2) random blood glucose ≥ 11.1 mmol/L; and 3) oral glucose tolerance test (OGTT) reading of ≥ 11.0 mmol/ L at 2 h. Non-diabetic participants were characterized by glycosylated hemoglobin levels between 4% and 5.6% and fasting blood glucose levels between 70 and 100 mg/dL.

### Laboratory measurements of plasma RBP4

Fasting blood samples were collected from physical examination with informed consent and immediately transported to the laboratory in an ice box. Plasma was obtained after centrifugation and frozen at − 80 °C until assay. The plasma RBP4 levels were measured using an enzyme-linked immunosorbent assay (ELISA) kit (JL24606, Jianglaibio Biotech, Shanghai, China), with the minimum detection limits and sensitivity values of 2 μg/mL and 0.1 μg/ml, respectively. The RBP4 assay demonstrated intra- and inter-assay variation coefficients of less than 9% and 11%, respectively. The absorbance value was measured at 450 nm by a multi-detection microplate reader (Thermo Scientific, USA). Considering the measurement error among batches, the standard curve was used to revise the data, and the same conditions and external environment were tried to maintain in each board.

### Covariates

In this study, covariates, including age, gender, education, family income (RMB/year), marital status, tobacco smoking, alcohol drinking and family history of diabetes, hypertension and coronary heart disease, were collected using a self- administered questionnaire. Height and weight information came from physical examination data. Education was divided into 'junior school', 'high school', and 'college and higher'; family income (RMB/year) was divided into ' ≤ 4000' and ' > 4000'; marital status was divided into 'cohabiting' and 'others'; tobacco smoking was divided into 'never', 'ever' and 'current'; alcohol drinking was divided into 'never', 'ever,' and 'current'; family history of diabetes, hypertension, and coronary heart disease was divided into "yes" and "no".

### Statistical analysis

SPSS for Windows, Version 22.0 (SPSS Inc., Chicago, IL, USA) was used for data processing and statistical analysis. The measurement data were statistically described as X ± S. Two independent samples t-tests were employed to compare the two groups. A chi-square test was conducted to compare the rate. Multiple logistic regression was used to analyze the associations of rotating night shifts and other factors with T2DM and RBP4. Multivariate linear mixed models were used to assess shift work's effect on changes in RBP4 concentration. The significance level was α = 0.05.

## Result

### Subject characteristics

Among 1499 cases, 762 (50.8%) worked in shifts. There were significant differences in age, sex, body mass index (BMI), family income, smoking, drinking, and diabetes prevalence between shift workers and day workers (*P* < 0.05). The mean age of shift workers was 40.31 years, lower than that of day workers (41.34 years). The proportion of females engaged in shift work (56.0%) was higher than that of male (45.7%). The BMI of shift workers (24.57) was slightly higher than that of day workers (24.19). The proportion of shift workers (72.8%) whose monthly family income was more than 4000 yuan was higher than that of day workers (64.5%). The proportion of shift workers with a smoking (30.4%) and drinking history (45.8%) was lower than that of day workers (36.9%, 54.7%). The prevalence of diabetes in shift workers (6.6%) was higher than in day workers (4.2%). There was no significant difference in education, marital status, and family history of diabetes, hypertension and royal heart disease patients (*P* > 0.05, Table 1). No statistically significant difference was found in diabetes prevalence among different type of shift work schedules (Supplementary Table 1).

**Table 1:** The characteristics of participants according to shift work status

Demographic	All subjects	Non-shift workers	shift workers	*P*-value
characteristics	(*n* = 1499)	(*n* = 737)	(*n* = 762)	
	Mean ± SD or n(%)	Mean ± SD or n(%)	Mean ± SD or n(%)	
Age (years)	40.82 ± 8.31	41.34 ± 8.36	40.31 ± 8.23	**0.017**
Sex				
male	751(50.1)	408(55.4)	343(45.0)	**< 0.001**
female	748(49.9)	329(44.6)	419(55.0)	
BMI	24.39 ± 3.59	24.19 ± 3.54	24.57 ± 3.64	**0.044**
Education level				
Primary school and below				
Junior school	1055(70.4)	508(68.9)	547(71.8)	0.285
High school	433(28.9)	225(30.5)	208(27.3)	
College and higher	11(0.7)	4(0.5)	7(0.9)	
Family income (RMB/monthly)				
≤ 4000	469(31.3)	262(35.5)	207(27.2)	**< 0.001**
> 4000	1030(68.7)	475(64.5)	555(72.8)	
Marital status				
cohabiting	1180(78.7)	588(79.8)	592(77.7)	0.322
other	319(21.3)	149(20.2)	170(22.3)	
Tobacco smoking				
Never	995(66.4)	465(63.1)	530(69.6)	**0.026**
Ever	150(10.0)	78(10.6)	72(9.4)	
Current	354(23.6)	194(26.3)	160(21.0)	
Alcohol drinking				
Never	747(49.8)	334(45.3)	413(54.2)	**< 0.001**
Ever	575(38.4)	297(40.3)	278(36.5)	
Current	117(11.8)	106(14.4)	71(9.3)	
Diabetes				
yes	81(5.4)	31(4.2)	50(6.6)	**0.044**
no	1418(94.6)	706(95.8)	712(93.4)	
Family history of diabetes/ hypertension/ coronary heart disease				
yes	400(27.2)	185(26.2)	215(28.2)	0.378
no	1099(72.8)	522(73.8)	547(71.8)	
PSQI global score	6.45 ± 3.15	5.99 ± 2.87	6.89 ± 3.35	**< 0.001**

### Difference between PSQI results of shift workers and day workers

Our study demonstrated significant differences between the two groups in the PSQI global score (Table 1), subjective sleep quality, sleep latency, sleep duration, habitual sleep efficiency, sleep disturbances, and daytime dysfunction (*P* < 0.05, Supplementary Table 2).

### Effect of shift work on the prevalence of T2DM

**Fig. 2:**
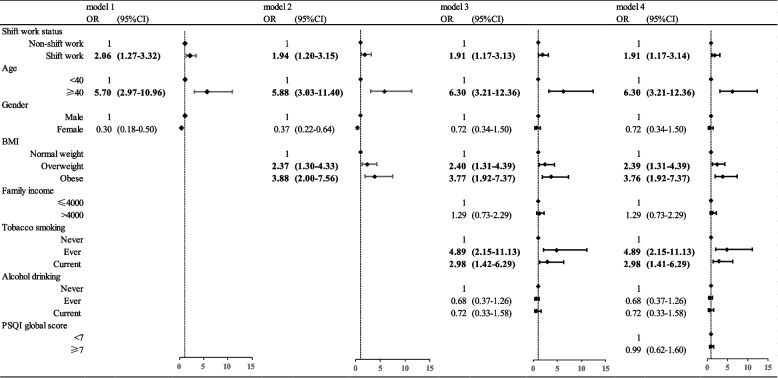
Multivariate logistic regression analyses investigating associations of rotating night shift and other factors with T2D among workers in China

Our study examined the impact of shift work on the prevalence of T2DM. A simple logistic regression analysis was carried out without considering other factors demonstrating 59.9% (OR:1.60; 95% CI:1.01–2.53, Supplementary Table 3) elevated risk in the shift work group compared to day shift work. The OR for the risk of T2DM in the shift group was 2.06 (95% CI:1.27 – 3.32) compared with the day shift group after adjusting for age and gender in Model 1. In Model 2, after adjusting for age, sex, and Body Mass Index (BMI), the shift work group displayed a 94.2%(OR:1. 94; 95% CI:1.20–3.15) increased risk of T2DM compared to the non-shift work group. Even in Model 3, the risk of T2DM was 91.2% higher in the shift group than in the non-shift group (OR:1.91; 95% CI:1.17 – 3.13) after adjusting for variables such as age, gender, BMI, family income, smoking, and alcohol consumption. Similarly, model 4 was adjusted for all univariate variables, demonstrating that the risk of T2DM in the shift group increased by 91.3% (OR:1.91; 95% CI:1.17–3.14) compared to the non-shift group (Fig. 2).

### Assessment of plasma RBP4 levels in different groups and the relationship between shift work exposure and RBP4 levels

**Fig. 3:**
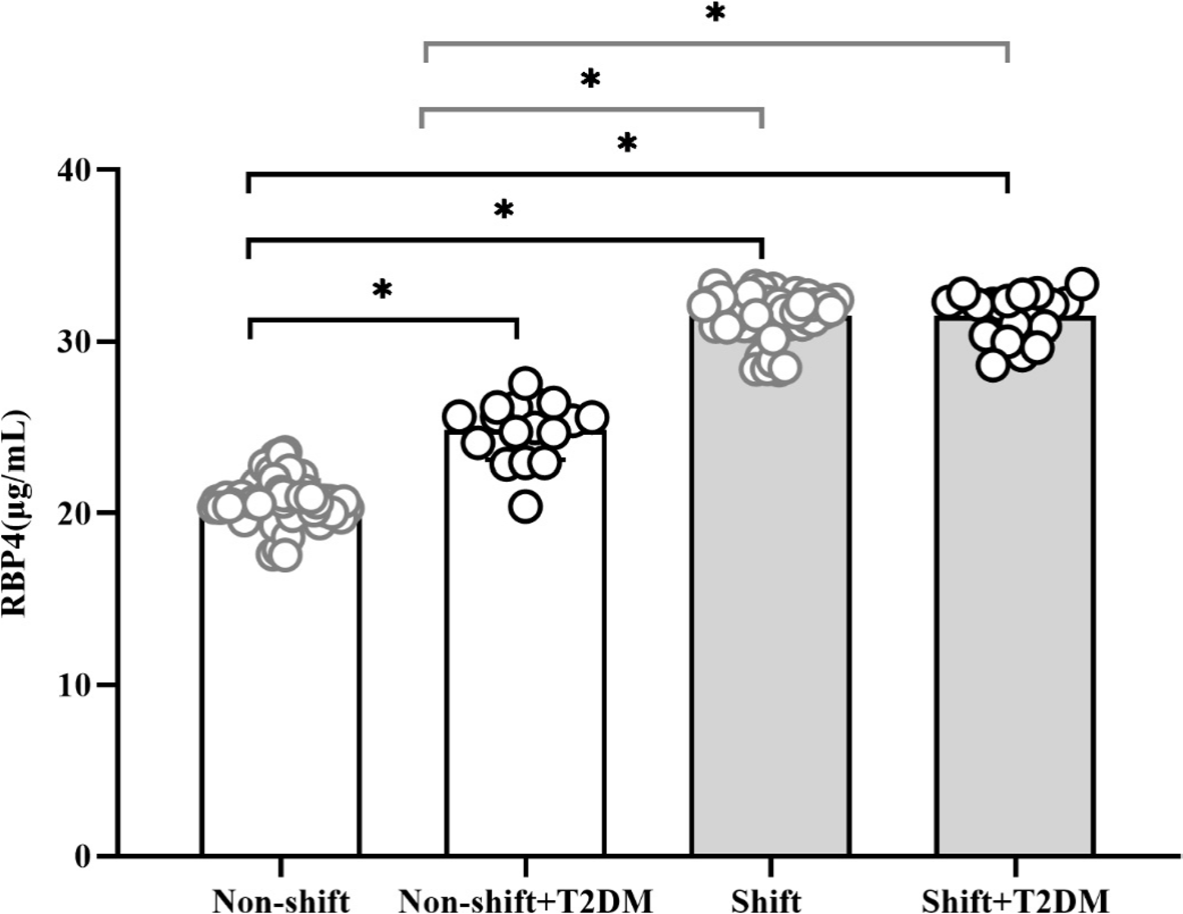
Comparison of RBP4 levels in study participants

To monitor the degree of the potential responses of RBP4 levels to shift exposure, we assessed serum RBP4 levels (Fig. 3). Compared to the day workers without T2DM, the shift workers without T2DM showed a 51.7% (31.50 ± 1.28 μg/mL vs. 20.77 ± 1.14 μg/mL) elevation in RBP4 concentration, and that of day workers with T2DM was 21.0% (25.12 ± 1.37 μg/mL vs. 20.77 ± 1.14 μg/mL). The RBP4 concentration was 25.4%(31.50 ± 1.28 μg/mL vs. 25.12 ± 1.37 μg/mL) and 25.5%(31.51 ± 1.36 μg/mL vs. 25.12 ± 1.37 μg/mL) higher in shift workers without T2DM and those with T2DM, respectively, compared to day workers with T2DM. However, there was no significant difference in the RBP4 levels across shift work schedules(Supplementary Fig. 1).

Finally, multivariate linear mixed models were utilized to assess shift work's effect on RBP4 concentration (Table 2). Shift work exposure significantly predicted RBP4 concentration outcomes across models adjusted for different variables. The RBP4 levels of shift workers increased by an average of 9.51 μg/mL compared with the day shift, when age, gender, BMI, diabetes, PSQI, family income, smoking and drinking remained unchanged in the last multivariate linear mixed models.

**Table 2:** Multivariate regression estimates of the effect of shift-work exposure on RBP4 after adjusting for covariates

Outcome variables	Partial regression coefficient	95%CI	*P*-value	Overall model fit
RBP4 ^a^	9.78	9.10–10.47	** < 0.001**	F = 265.14, R2 = 0.88, ***p* < 0.001**
RBP4 ^b^	9.42	8.83–10.02	** < 0.001**	F = 298.24, R2 = 0.91, ***p* < 0.001**
RBP4 ^c^	9.51	8.92–10.11	** < 0.001**	F = 190.66, R2 = 0.91, ***p* < 0.001**

In addition to shift work exposedness, models were adjusted for:

^a^ adjusted for age, sex and BMI

^b^ adjusted for age, sex, BMI and diabetes

^c^ adjusted for age, sex, BMI, diabetes, PSQI, family income, tobacco smoking and alcohol drinking

## Discussion

This study investigated the potential relationship between shift work and T2DM with the RBP4 level. Our results affirm earlier findings that shift workers had a 106% (OR = 2.06, 95%CI:1.27–3.32) increased risk of T2DM compared to non-shift workers. The association remained persistent even after full adjustment for multiple potential confounding factors. Diverging from past research, we noticed a correlation between shift work and RBP4 levels. Specifically, RBP4 levels in shift workers exceeded those in non-shift workers, with shift work exposure serving as a significant predictor of the RBP4 concentration throughout the models adjusted for different variables.

Diabetes was the most common disease reported by shift workers [22]. Shift workers needed to rotate in different shifts, resulting in a certain "conflict" between working and social time. This disturbed the normal biological rhythm, led to changes in sleep, diet, exercise habits, etc., and disturbed the normal glucose and lipid metabolism. At the same time, the accumulation of shift time and the frequent occurrence of biological rhythm disorders, glucose homeostasis imbalance, and IR eventually culminate in chronic diseases such as T2DM.

The Atlantic Path Cohort study showed that shift workers were 27% more likely to develop diabetes than non-shift workers (95%CI: 8–51) [23]. Hansen [9] et al. followed up 19,873 nurses in Denmark for 15 years to study the relationship between shift work and diabetes incidence rate. They found that after adjusting BMI, the risk of nurses who had diabetes on the night shift was 1.58(95% *CI*: 1.25–1.99) times to those on the day shift. A meta-analysis based on 38 meta-analyses and 24 systematic reviews reported a meta-relative risk ranging from 1.09 to 1.40 in shift workers [24]. This study found that the risk of diabetes among shift workers was 1.60(95% CI: 1.01–2.53) times that of day workers, as evidenced by investigating the relationship between shift work and the risk of T2DM among oil workers in western China.

The population, gender, BMI and living habits of shift workers would impact the incidence of T2DM. There were population [25] and gender [26] differences between shift work and the risk of T2DM. Shift workers were more likely to be overweight than day shift workers [27]. Adverse health behaviors caused by shift work, such as changes in living habits, smoking, and poor sleep quality [28], coupled with the destruction of biological rhythm, could produce adverse metabolic phenotypes. In this study, shift work of oil workers in western China was related to the risk of T2DM in the models adjusting for different variables. Overweight and (or) obesity was one of the influencing factors of T2DM. Adjusting for age, gender, and BMI demonstrated that shift workers were 94.2%(95% *CI*: 1.20–3.15) more likely to develop diabetes. Further adjusting for age, gender, BMI, smoking, drinking, and family income, our study found that shift work was still a risk factor for diabetes (OR = 1.91, 95% *CI*: 1.17–3.13).

Shift workers' non-conventional hours, misaligned with their physiological and social circadian rhythm, predispose them to sleep disorders, such as excessive sleepiness and/or insomnia [29, 30]. This study further demonstrated that shift work had a negative impact on sleep quality. Although the subjects had different degrees of sleep disorders, compared with the non-shift work group, the total PSQI score of the reverse group was higher, indicating that the sleep disorders were more serious. Zhang et al. [31] found that current and previous shifts were major factors affecting sleep quality. They found that the current shift work was significantly correlated with sleep efficiency, sleep quality, and daily dysfunction. Park et al. [32] found that the PSQI score of shift workers was significantly higher than day workers. Similar findings by Zhang et al. [33] reported a higher prevalence of sleep disorders in shift workers than in day workers. A cohort study found an elevated risk of sleep disorders in individuals with irregular working hours compared with daytime work [34]. External and environmental factors largely preserve the synchronization of the circadian rhythm to the 24-h cycle. However, shift work interferes with natural sleep–wake rhythms, leading to conflicts between endogenous sleep and wakefulness. This discordance significantly impacts both sleep and wakefulness negatively. Shift workers need to stay awake and work at night when the circadian alarm signal is the lowest and sleepiness is the most serious, often in the face of minimal night-time stimuli [35].

Sleep is a major part of the lifestyle. Controlled laboratory studies have shown that sleep restriction could lead to impaired glucose tolerance [36], closely related to the onset of diabetes. However, our study did not find significant evidence linking sleep impact with diabetes prevalence during shift work, potentially due to varied sleep disorder rates among participants (because the data are meaningless and not listed).

RBP4 promotes the occurrence of IR in tissues and organs by influencing glucose uptake and the regulation of gluconeogenesis. Thus RBP4 plays a key role in the occurrence and development of diabetes and other related diseases. Bose et al [37]. found that higher serum RBP-4 had a positive correlation with Insulin, glucose, and HOMA-IR in healthy non-diabetic participants with a family history of diabetes. In a 10-year prospective study, it was found that NGT with higher serum RBP4 was associated with new-onset prediabetes and T2DM [38]. Furthermore, animal studies showed that GLUT4 knockout in adipose tissue triggers RBP4 overexpression, promoting T2DM onset [39]. Laparoscopic sleeve gastrectomy may bring a decline in the level of RBP4 and improve IR in obese patients [40]. RBP4 could alter downstream gene expression and signal transduction pathways through STRA6 -JAK/STAT [41] and MAPK signal transduction pathway, finally inducing IR and accelerating the production of T2DM [17–20]. Norseen et al. [19] and Moraes Vieira et al. [42, 43] found that RBP4 could induce IR through immune-related pathways. Besides, RBP4 may have a potential role in the pathogenesis of diabetic complications such as diabetic atherosclerosis [44] and diabetic nephropathy [45].

We measured the levels of RBP4 in different studies and revealed shift exposure as a vital risk factor for changes in RBP4 concentration in models adjusted for different variables. Among the participants of the same age, gender, BMI, diabetes, PSQI, family income, smoking and drinking, the RBP4 level of shift workers increased by an average of 9.51 μg/mL compared with the non-shift workers. To our knowledge, this is the first research on the relationship between shift work and RBP4 among oil workers. RBP4 might be a predictor for the onset of diabetes in shift workers in the coming future. Ma et al. [21] proposed that RBP4 was a gene controlled by circadian rhythm. The circadian clock gene BMAL1 in mouse liver drives RBP4 to regulate glucose metabolism through its direct target DBP. This discovery not only provided a new mechanism for the circadian regulation of RBP4, but also unveiled the key role of RBP4 in regulating glucose metabolism through the biological clock, as well as the time-dependent association between RBP4 and mouse IR, which had potential importance in the clinical prognosis of T2DM and metabolic syndrome with IR. Further studies are warranted to validate these findings.

However, no statistically significant difference was observed in RBP4 levels between shift workers with diabetes and those without diabetes in our study. This might be attributable to factors such as sample size and sampling methods. On the other hand, the improved RBP4 levels among diabetic shift workers could stem from social support and education provided by hospitals, communities, and families and the use of hypoglycemic drugs [46]. These findings require future validation.

The study has limitations that should be addressed in future research. First, we can only describe the relationships among shift work, RBP4, and T2DM through a cross-sectional study, but we cannot explain the temporality and casual relationships among the three. Understanding the correlation between work-related environment and diabetes biomarkers could enhance the effectiveness of intervention and prevention strategies to improve the working conditions and the health of individual workers. Second, the covariates, shift work and sleep quality were obtained through the self-report questionnaire, which- can increase the risk of misclassification bias. In the future, actual work records can be used to identify shift work schedules. Besides the international PSQI scale used in the measurement of sleep disorders, other tools like sleep diaries, activity recorders, and the Bergen shift work sleep questionnaire (BSWSQ) and the shift work disorder questionnaire (SWDQ)—geared towards evaluating insomnia and sleepiness symptoms associated with shift work sleep disorders could be considered to reduce subjective impact. Third, currently, participants complete the PSQI solely during corporate health examinations, calling for a more standardized schedule, specifying whether it's during night or day shifts, before night shift commencement, or after the first-day shift rest. It should be unified or clear when workers will complete the questionnaire survey (during the night shift or night shift, before the start of the night shift, or after the first-day shift rest). Finally, some confounding factors were not addressed in this study. Co-exposure to volatile aromatic hydrocarbons such as BTEX, styrene and noise is common among oil workers, with past studies suggesting a potential link between these factors and diabetes risk [47, 48]. Possible confounding factors should be further controlled in the future.

More comprehensive studies, particularly cohort studies, are needed to explore other potential risk factors linking shift work with RBP4 and T2DM, aiding in mitigating diabetes risk among shift workers. Therefore, the findings of our study should be regarded as preliminary, necessitating further research in this field.

## Conclusions

Our study showed that shift work was associated with increased RBP4 levels and was a hazard factor for T2DM. Our findings propose a potential role for RBP4 in the pathogenesis of T2DM, contributing to a heightened risk among shift workers. Future studies with many available clinical specimens and functional experiments are needed to verify these findings and better expound the underlying mechanisms.

## Supplementary Information

**Additional file 1: Supplementary Table 1.** Characteristics of participants according to the different working shifts.** Supplementary Table 2.** The mean PSQI component according to shift work status.** Supplementary Table 3.** The Logistic regression analyses investigating associations of shift and other factors with T2D among workers in China.

**Additional file 2: Supplementary Figure 1.** Comparison of RBP4 levels in different shift work schedules.

## Data Availability

The datasets used and analyzed during the current study are available from the corresponding author on reasonable request.

## Abbreviations

T2DM: Type 2 diabetes
RBP4: Retinol binding protein 4
IARC: International Agency for Research on Cancer
IR: Insulin resistance
SNP: Single-nucleotide polymorphism
IRS: Insulin receptor substrate
MAPK: Mitogen activated protein kinase
JNK: C-Jun N-terminal kinase
OHSPIW: Occupational Health Study of Petroleum Industry Workers
CNPC: China National Petroleum Corporation
PSQI: Pittsburgh Sleep Quality Index
WHO: World Health Organization
OGTT: Oral glucose tolerance test
BMI: Body mass index
BSWSQ: Bergen shift work sleep questionnaire
SWDQ: Shift work disorder questionnaire

## References

[1] Eurofound. Sixth European working conditions survey – overview report. 2016. https://hdl.handle.net/1813/87536. Accessed 1 Jan 2016.

[2] Maestas N, Mullen K, Powell D, Wachter T, Wenger J. Working conditions in the United States: results of the 2015 American working conditions survey. 2017. https://www.rand.org/pubs/research_reports/RR2014.html. Accessed 4 Aug 2017.

[3] Suwazono Y, Dochi M, Sakata K (2008). A longitudinal study on the effect of shift work on weight gain in male Japanese workers. Obesity.

[4] Ward EM, Germolec D, Kogevinas M (2019). Carcinogenicity of night shift work. Lancet Oncol.

[5] Jagannath A, Taylor L, Wakaf Z (2017). The genetics of circadian rhythms, sleep and health. Hum Mole Gen.

[6] Hernández-García J, Navas-Carrillo D, Orenes-Piñero E. Alterations of circadian rhythms and their impact on obesity, metabolic syndrome and cardiovascular diseases. Crit Rev Food Sci Nutr 2019:1–10. 10.1080/10408398.2018.1556579 30633544

[7] Alachkar A, Lee J, Asthana K (2022). The hidden link between circadian entropy and mental health disorders. Transl Psychiatry.

[8] Bragg F, Holmes MV, Iona A (2017). Association between diabetes and cause-specific mortality in rural and urban areas of China. JAMA, J Am Med Assoc.

[9] Hansen AB, Stayner L, Hansen J (2016). Night shift work and incidence of diabetes in the Danish nurse cohort. Occup Environ Med.

[10] Itani O, Kaneita Y, Tokiya M (2017). Short sleep duration, shift work, and actual days taken off work are predictive life-style risk factors for new-onset metabolic syndrome: a seven-year cohort study of 40,000 male workers. Sleep Med.

[11] Quadro L, Blaner WS, Hamberger L (2004). The role of extrahepatic retinol binding protein in the mobilization of retinoid stores. J Lipid Res.

[12] Yang Q, Graham T, Mody N (2005). Serum retinol binding protein 4 contributes to insulin resistance in obesity and type 2 diabetes. Nature.

[13] Graham TE, Yang Q, Blüher M (2006). Retinol-binding protein 4 and insulin resistance in lean, obese, and diabetic subjects. N Engl J Med.

[14] Cho YM, Youn BS, Lee H (2006). Plasma retinol-binding protein-4 concentrations are elevated in human subjects with impaired glucose tolerance and type 2 diabetes. Am Diab Assoc.

[15] Klting N, Fasshauer M, Dietrich A (2010). Insulin-sensitive obesity. AJP Endocrinol Metab.

[16] Janke J, Engeli S, Boschmann M (2006). Retinol-binding protein 4 in human obesity. Diabetes.

[17] Michaela V, Eva K, Michaela K (2007). Plasma levels and adipose tissue messenger ribonucleic acid expression of retinol-binding protein 4 are reduced during calorie restriction in obese subjects but are not related to diet-induced changes in insulin sensitivity. J Clin Endocrinol Metab.

[18] Kunjathoor V, Febbraio M, Podrez E (2002). Scavenger receptors class A-I/II and CD36 are the principal receptors responsible for the uptake of modified low density lipoprotein leading to lipid loading in macrophages. J Biol Chem.

[19] Norseen J, Hosooka T, Hammarstedt A (2012). Retinol-binding protein 4 inhibits insulin signaling in adipocytes by inducing proinflammatory cytokines in macrophages through a c-Jun N-terminal kinase- and toll-like receptor 4-dependent and retinol-independent mechanism. Mol Cell Biol.

[20] Berry D, Jin H, Majumdar A (2011). Signaling by vitamin A and retinol-binding protein regulates gene expression to inhibit insulin responses. Proc Natl Acad Sci USA.

[21] Ma X, Zhou Z, Chen Y (2016). RBP4 functions as a hepatokine in the regulation of glucose metabolism by the circadian clock in mice. Diabetologia.

[22] Nena E, Katsaouni M, Steiropoulos P (2018). Effect of Shift Work on Sleep, Health, and Quality of Life of Health-care Workers. Indian J Occup Environ Med.

[23] Sweeney E, Yu Z, Dummer T (2020). The relationship between anthropometric measures and cardiometabolic health in shift work: findings from the Atlantic PATH Cohort Study. Int Arch Occup Environ Health.

[24] Kecklund G, Axelsson J (2016). Health consequences of shift work and insufficient sleep. BMJ (Clinical Research ed).

[25] Night-shift work linked to diabetes risk in black women. Nursing standard (Royal College of Nursing (Great Britain):1987) 2015;29(20):10 10.7748/ns.29.20.10.s10 25585728

[26] Hanprathet N, Lertmaharit S, Lohsoonthorn V (2019). Increased risk of type 2 diabetes and abnormal FPG due to shift work differs according to gender: a retrospective cohort study among Thai workers in Bangkok, Thailand. Diab Metab Syndr Obes : Targets Ther.

[27] Hulsegge G, Picavet HSJ, van der Beek AJ (2019). Shift work, chronotype and the risk of cardiometabolic risk factors. Eur J Public Health.

[28] Yanxia Z: Prospective cohort study on the relationship between occupational stress and sleep quality of petroleum workers. D. Xinjiang Medical University; 2015.

[29] Huang L, Tsai M, Chen C (2013). The effectiveness of light/dark exposure to treat insomnia in female nurses undertaking shift work during the evening/night shift. J Clin Sleep Med : JCSM : Public Am Acad Sleep Med.

[30] Sun Q, Ji X, Zhou W (2019). Sleep problems in shift nurses: A brief review and recommendations at both individual and institutional levels. J Nurs Manag.

[31] Zhang L, Sun D, Li C (2016). Influencing factors for sleep quality among shift-working nurses: a cross-sectional study in China using 3-factor Pittsburgh sleep quality index. Asian Nurs Res.

[32] Park H, Suh B, Lee S (2019). Shift work and depressive symptoms: the mediating effect of vitamin D and sleep quality. Chronobiol Int.

[33] Zhang Y, Shen J, Zhou Z (2020). Relationships among shift work, hair cortisol concentration and sleep disorders: a cross-sectional study in China. BMJ open.

[34] Rugulies R, Norborg M, Sørensen T (2009). Effort-reward imbalance at work and risk of sleep disturbances cross-sectional and prospective results from the Danish work environment cohort study. J Psychosom Res.

[35] Wickwire E, Geiger-Brown J, Scharf S (2017). Shift work and shift work sleep disorder: clinical and organizational perspectives. Chest.

[36] Spiegel KLR, Van Cauter E (1999). Impact of sleep debt on metabolic and endocrine function. Lancet.

[37] Bose KS, Gupta SK, Singh S (2012). Is serum retinol binding protein-4: A predictor for diabetes in genetically high risk population?. J Res Med Sci.

[38] Cho N, Ku E, Jung K (2020). Estimated association between cytokines and the progression to diabetes: 10-year follow-up from a community-based cohort. J Clin Endocrinol Metab.

[39] Yamauchi J, Sekiguchi M, Shirai T (2013). Role of nuclear localization of PSMB1 in transcriptional activation. Biosci Biotechnol Biochem.

[40] Wang X, Huang Y, Gao J (2020). Changes of serum retinol-binding protein 4 associated with improved insulin resistance after laparoscopic sleeve gastrectomy in Chinese obese patients. Diabetol Metab Syndr.

[41] Gliniak C, Brown J, Noy N (2017). The retinol-binding protein receptor STRA6 regulates diurnal insulin responses. J Biol Chem.

[42] Moraes-Vieira P, Yore M, Dwyer P (2014). RBP4 activates antigen-presenting cells, leading to adipose tissue inflammation and systemic insulin resistance. Cell Metab.

[43] Moraes-Vieira P, Castoldi A, Aryal P (2016). Antigen presentation and T-cell activation are critical for RBP4-induced insulin resistance. Diabetes.

[44] Zhou W, Ye S, Wang W (2021). viaElevated retinol binding protein 4 levels are associated with atherosclerosis in diabetic rats JAK2/STAT3 signaling pathway. World J Diab.

[45] Zhang L, Cheng Y, Xue S (2020). The role of circulating RBP4 in the type 2 diabetes patients with kidney diseases: a systematic review and meta-analysis. Dis Markers.

[46] Li R, Yang W, Xie R (2022). Clinical study on Yangyin Jiangtang tablets combined with glimepiride in treatment of type 2 diabetes. Drugs Clin.

[47] Lee I, Park H, Kim MJ (2022). Exposure to polycyclic aromatic hydrocarbons and volatile organic compounds is associated with a risk of obesity and diabetes mellitus among Korean adults: Korean National Environmental Health Survey (KoNEHS) 2015–2017. Int J Hyg Environ Health.

[48] Yu L, Liu W, Zhou M (2023). Long-term effect of styrene and ethylbenzene exposure on fasting plasma glucose: a gene-environment interaction study. J Hazard Mat.

