# Supplementary material for: Shift work is associated with an increased risk of type 2 diabetes and elevated RBP4 level: cross sectional analysis from the OHSPIW cohort study
Source: BMC Public Health. 2023 Jun 14;23:1139. doi: 10.1186/s12889-023-16091-y (PMC10265876; doi:10.1186/s12889-023-16091-y)
Supplement: Supplementary file 1 — Additional file 1: Supplementary Table 1. Characteristics of participants according to the different working shifts. Supplementary Table 2. The mean PSQI component according to shift work status. Supplementary Table 3. The Logistic regression analyses investigating associations of shift and other factors with T2D among workers in China. [file 12889_2023_16091_MOESM1_ESM.docx]

Supplementary Table 1 Characteristics of participants according to

the different working shifts

| Demographic | Day shift | two shifts | three shifts | four shifts | others | P-value |
| --- | --- | --- | --- | --- | --- | --- |
| characteristics | (n=1499) | (n=303) | (n=91) | (n=267) | (n=101) |  |
|  | Mean±SD or n(%) | Mean±SD or n(%) | Mean±SD or n(%) | Mean±SD or n(%) | Mean±SD or n(%) |  |
| Age (years) | 41.34±8.36 | 39.63±8.74 | 40.60±8.62 | 41.51±7.29 | 38.91±8.37 | **0.003** |
| Sex |  |  |  |  |  |  |
| male | 408(55.4) | 166(54.8) | 39(42.9) | 85(31.8) | 53(52.5) | **<0.001** |
| female | 329(44.6) | 137(45.2) | 52(57.1) | 182(68.2) | 48(47.5) |  |
| BMI | 24.19±3.54 | 24.84±3.47 | 24.49±3.58 | 24.24±3.72 | 24.69±3.94 | 0.087 |
| Education level |  |  |  |  |  |  |
| Junior school | 508(68.9) | 228(75.2) | 70(76.9) | 185(69.3) | 64(63.4) | 0.072 |
| High school and higher | 229(31.1) | 75(24.8) | 21(23.1) | 82(30.7) | 37(36.6) |  |
| Family income (RMB/monthly) |  |  |  |  |  |  |
| ≤4000 | 262(35.5) | 72(23.8) | 26(28.6) | 75(28.1) | 34(33.7) | **0.003** |
| >4000 | 475(64.5) | 231(76.2) | 65(71.4) | 192(71.9) | 67(66.3) |  |
| Marital status |  |  |  |  |  |  |
| cohabiting | 588(79.8) | 229(75.6) | 64(70.3) | 220(82.4) | 79(78.2) | 0.082 |
| other | 149(20.2) | 74(24.4) | 27(29.7) | 47(17.6) | 22(21.8) |  |
| Tobacco smoking |  |  |  |  |  |  |
| Never | 465(63.1) | 194(64.0) | 62(68.1) | 209(78.3) | 65(64.4) | **0.002** |
| Ever | 78(10.6) | 32(10.6) | 7(7.7) | 19(7.1) | 14(13.9) |  |
| Current | 194(26.3) | 77(25.4) | 22(24.2) | 39(14.6) | 22(21.8) |  |
| Alcohol drinking |  |  |  |  |  |  |
| Never | 334(45.3) | 148(48.8) | 52(57.1) | 165(61.8) | 48(47.5) | **<0.001** |
| Ever | 297(40.3) | 125(41.3) | 27(29.7) | 82(30.7) | 44(43.6) |  |
| Current | 106(14.4) | 30(9.9) | 12(13.2) | 20(7.5) | 9(8.9) |  |
| Diabetes |  |  |  |  |  |  |
| yes | 31(4.2) | 21(6.9) | 4(4.4) | 17(6.4) | 8(7.9) | 0.219 |
| no | 706(95.8) | 282(93.1) | 87(95.6) | 250(93.6) | 93(92.1) |  |
| Family history of diabetes/ hypertension/ coronary heart disease |  |  |  |  |  |  |
| yes | 185(26.2) | 75(24.8) | 25(27.5) | 83(31.1) | 32(31.7) | 0.360 |
| no | 522(73.8) | 228(75.2) | 66(72.5) | 184(68.9) | 69(68.3) |  |
| PSQI global score | 5.99±2.87 | 6.84±3.20 | 6.01±2.83 | 7.07±3.55 | 7.37±3.58 | **<0.001** |

Supplementary Table 2 The mean PSQI component according to shift work status

| Variables for PSQI | All subjects | Non-shift workers | shift workers | P-value |
| --- | --- | --- | --- | --- |
|  | (n=1499) | (n=737) | (n=762) |  |
|  | Mean score±SD | Mean score±SD | Mean score±SD |  |
| Subjective sleep quality | 1.24±0.75 | 1.15±0.70 | 1.32±0.78 | **<0.001** |
| Sleep latency | 1.33±0.92 | 1.19±0.89 | 1.48±0.92 | **<0.001** |
| Sleep duration | 0.91±0.61 | 0.86±0.61 | 0.96±0.62 | **0.003** |
| Habitual sleep efficiency | 0.26±0.63 | 0.22±0.59 | 0.31±0.67 | **0.007** |
| Sleep disturbances | 1.06±0.57 | 1.00±0.51 | 1.12±0.61 | **<0.001** |
| Use of sleep medication | 0.22±0.61 | 0.21±0.59 | 0.24±0.63 | 0.408 |
| Daytime dysfunction | 1.42±0.86 | 1.36±0.81 | 1.47±0.90 | **0.013** |

Supplementary Table 3 The Logistic regression analyses investigating associations of shift and other factors with T2D among workers in China

|  | OR | (95%CI) | P |
| --- | --- | --- | --- |
|  |  |  |  |
| Shift work status |  |  |  |
| Non-shift work | 1 |  |  |
| Shift work | **1.60** | **(1.01-2.53)** | **0.045** |
| Age |  |  |  |
| <40 | 1 |  |  |
| ≥40 | **4.90** | **(2.57-9.34)** | **<0.001** |
| Gender |  |  |  |
| Male | 1 |  |  |
| Female | **0.38** | **(0.23-0.62)** | **<0.001** |
| BMI |  |  |  |
| Normal weight | 1 |  |  |
| Overweight | **3.38** | **(1.90-6.01)** | **<0.001** |
| Obese | **4.64** | **(2.43-8.86)** | **<0.001** |
| Family income |  |  |  |
| ≤4000 | 1 |  |  |
| >4000 | **1.76** | **(1.02-3.04)** | **0.042** |
| Tobacco smoking |  |  |  |
| Never | 1 |  |  |
| Ever | **3.85** | **(2.08-7.12)** | **<0.001** |
| Current | **2.99** | **(1.80-4.96)** | **<0.001** |
| Alcohol drinking |  |  |  |
| Never | 1 |  |  |
| Ever | 1.32 | (0.80-2.16) | 0.275 |
| Current | **2.00** | **(1.06-3.78)** | **0.032** |
| PSQI global score |  |  |  |
| <7 | 1 |  |  |
| ≥7 | 1.05 | (0.67-1.65) | 0.830 |
