# Supplementary figures and images for: Shift work is associated with an increased risk of type 2 diabetes and elevated RBP4 level: cross sectional analysis from the OHSPIW cohort study
Source: BMC Public Health. 2023 Jun 14;23:1139. doi: 10.1186/s12889-023-16091-y (PMC10265876; doi:10.1186/s12889-023-16091-y)

## Slide 1
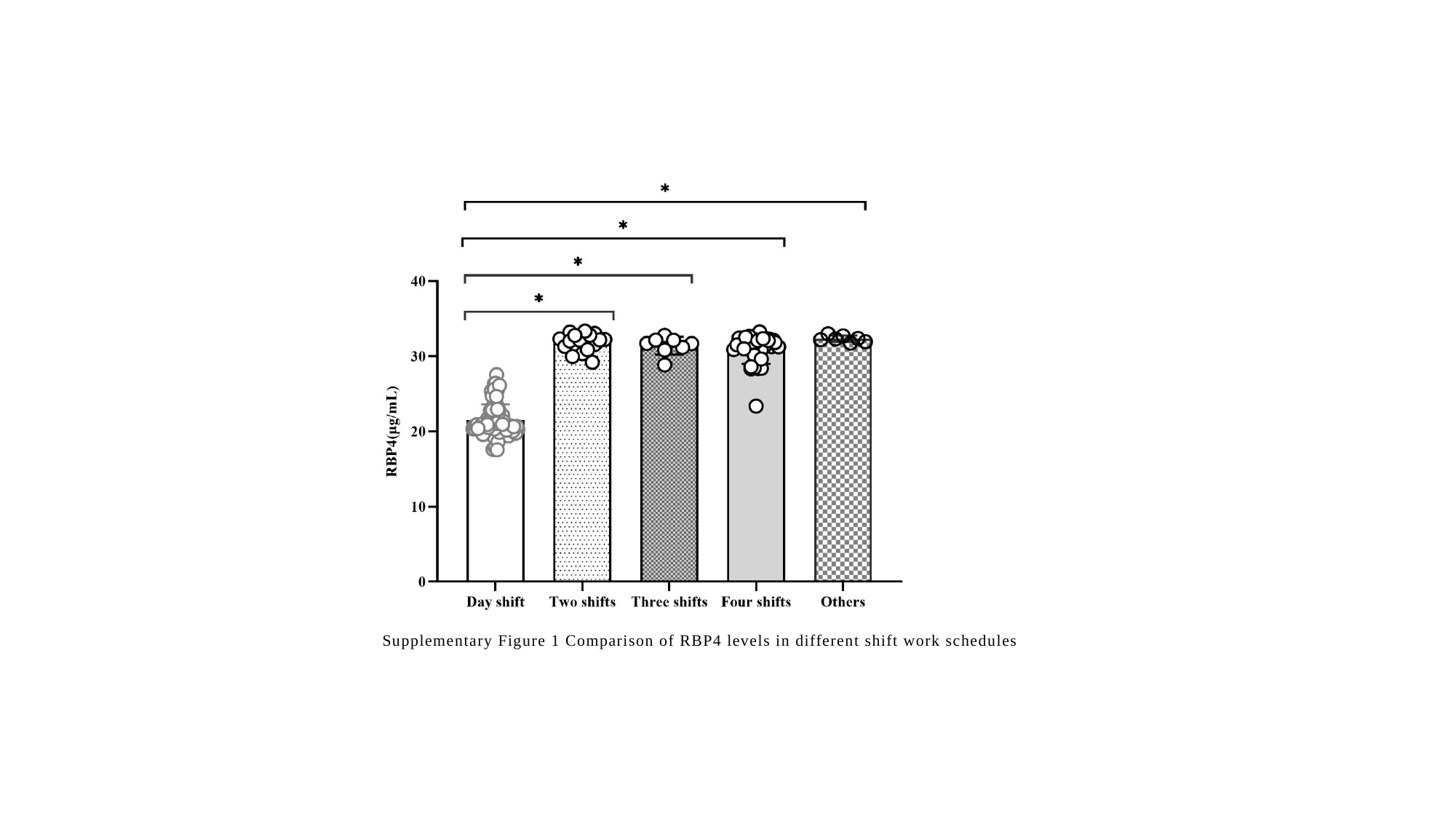

Supplementary Figure 1 Comparison of RBP4 levels in different shift work schedules

Supplement: Supplementary file 2 — Additional file 2: Supplementary Figure 1. Comparison of RBP4 levels in different shift work schedules. [file 12889_2023_16091_MOESM2_ESM.pptx]
